# Meta-analysis of human methylation data for evidence of sex-specific autosomal patterns

**DOI:** 10.1186/1471-2164-15-981

**Published:** 2014-11-18

**Authors:** Nina S McCarthy, Phillip E Melton, Gemma Cadby, Seyhan Yazar, Maria Franchina, Eric K Moses, David A Mackey, Alex W Hewitt

**Affiliations:** Centre for the Genetic Origins of Health and Disease (GOHaD), University of Western Australia, Perth, Australia; Centre for Ophthalmology and Vision Science, University of Western Australia and the Lions Eye Institute, Perth, Australia; Centre for Eye Research Australia, Royal Victorian Eye and Ear Hospital, University of Melbourne, Melbourne, Australia

**Keywords:** Methylation, Genome, Sex, CpG, Illumina Infinium HumanMethylation27K, Meta- analysis

## Abstract

**Background:**

Several individual studies have suggested that autosomal CpG methylation differs by sex both in terms of individual CpG sites and global autosomal CpG methylation. However, these findings have been inconsistent and plagued by spurious associations due to the cross reactivity of CpG probes on commercial microarrays. We collectively analysed 76 published studies (n = 6,795) for sex-associated differences in both autosomal and sex chromosome CpG sites.

**Results:**

Overall autosomal methylation profiles varied substantially by study, and we encountered substantial batch effects. We accounted for these by conducting random effects meta-analysis for individual autosomal CpG methylation associations. After excluding non-specific probes, we found 184 autosomal CpG sites differentially methylated by sex after correction for multiple testing. In line with previous studies, average beta differences were small. Many of the most significantly associated CpG probes were new. Of note was differential CpG methylation in the promoters of genes thought to be involved in spermatogenesis and male fertility, such as *SLC9A2*, *SPESP1*, *CRISP2*, and *NUPL1*. Pathway analysis revealed overrepresentation of genes differentially methylated by sex in several broad Gene Ontology biological processes, including RNA splicing and DNA repair.

**Conclusions:**

This study represents a comprehensive analysis of sex-specific methylation patterns. We demonstrate the existence of sex-specific methylation profiles and report a large number of novel DNA methylation differences in autosomal CpG sites between sexes.

**Electronic supplementary material:**

The online version of this article (doi:10.1186/1471-2164-15-981) contains supplementary material, which is available to authorized users.

## Background

DNA methylation of the eukaryotic genome is essential for normal cellular differentiation and embryonic development [1, 2]. Methylation within gene promoter regions is important in the regulation of gene expression [3], and changes in both overall methylation and specific methylation patterns have been shown to vary within an individual according to tissue type and disease status [4]. In healthy people, inter-individual differences in methylation are also observed and it has been postulated that these differences may be influenced by various factors, including sex [5–16].

Studies that have investigated methylation differences between males and females in repeat sequences (LINE and Alu) and other targeted genomic areas have reported sex-specific methylation differences at various autosomal sites and suggested a tendency toward higher methylation levels in males than in females [5–11], although one study reported no significant difference [17].

Advances in microarray technology have enabled the assessment of genome-wide methylation by surveying CpG methylation at thousands of sites across the genome. The Illumina Infinium series is a commonly used commercial platform and the HumanMethylation27K was one of the first comprehensive methylation microarrays. It interrogates over 27,500 CpG sites in the promoter regions of more than 14,000 RefSeq genes. Studies using this array have reported that a large number of autosomal CpG sites (up to 5% of autosomal loci, or 1,333 CpGs) appear to be differentially methylated in females and males, and that global CpG methylation is higher in males than in females [10, 12–16].

Recent work has demonstrated substantial sequence overlap between autosomal and sex-linked probes on the HumanMethylation27K microarray and that up to 10% of the probes are nonspecific and map to highly homologous genomic sequences [18]. The majority of the sex-associated methylation sites at autosomal CpG loci reported to date are likely to be technical artefacts created by the presence of cross-reactive autosomal probes hybridizing to both autosomal and sex chromosomes [18, 19].

We report new CpG associations with sex that are not due to nonspecific probes on the array. Many of the CpG associations lie in genes thought to be involved in spermatogenesis and male fertility, such as *SLC9A2*, *SPESP1*, *CRISP2*, and *NUPL1*. Pathway analysis revealed overrepresentation of genes differentially methylated between males and females in several Gene Ontology (GO) biological processes, such as regulation of immune response, RNA splicing and DNA repair. Our findings support previously reported global methylation differences between females and males: increased autosomal methylation in males and increased X chromosome methylation in females.

## Results

### X chromosome analysis and sex assignment

Following quality control (see Methods section), the final dataset included data on 7,333 samples from 81 studies from the European Bioinformatics Institute (EBI) database. Of these, 5,207 samples had sex recorded from the EBI phenotype files. The characteristics of this final dataset are displayed in Table 1.

**Table 1:** **Characteristics of the final dataset** (**n** = **7**,**333**, **81 studies**) **included in the analysis**

Study characteristics		
Mean study sample size (sd; range)		90.5 (120.0; 6-719)
Sex:	number recorded (%)	5,207 (71.0)
	-of which n female (%)	2,870 (55.1)
	n male (%)	2,277 (43.7)
	n other (trisomies/XXY) (%)	60 (1.1)
	following final classification:	
	n female (%)	3,647 (49.7)
	n male (%)	3,686 (50.3)
Sample Source:		
	n PBLs (%)	3,416 (46.6)
	n tissue (%)	3,917 (53.4)
	-of which n cancer tissue (%)	2,432 (62.1)

*Abbreviations:* *PBL* peripheral blood leukocytes, *n* number, *SD* standard deviation.

**Figure 1:**
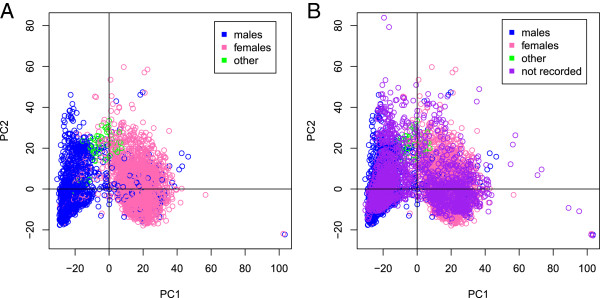
**Plot of the first two principal components (PC1 and PC2) based on the 999 X chromosome CpG sites for known males (n = 2,277), females (n = 2,870)** **and other (n = 60) (plot A) and with those of unknown sex (n = 2,126) superimposed (plot B).**

We initially sought to investigate whether sex could be inferred from X chromosome methylation data using principal component analysis (PCA). The first two principal components (PCs) were plotted against each other for all samples of known sex (Figure 1(A)). Colouring by the recorded sex from the EBI phenotype files indicates that sex can be determined by classifying samples based on their first PC, with samples recorded as ‘other/trisomy’ (n = 60) clustering in the middle. The second PC, in contrast, contributes little to the separation of males and females. Figure 1(B) shows that the samples of unknown sex cluster well with those of known sex. Logistic regression of recorded sex on PC1 and recorded sex on PC2 showed that both relationships were significant and, as expected, PC1 was a much better predictor than PC2 (PC1: *P* < 2e-16, AIC 2447.4; PC2: *P* = 1e-08, AIC 7037.2).

Due to X-inactivation as a result of lyonisation [13], global methylation values across the 999 X chromosome sites contained on the HumanMethylation27K BeadChip were expected to be higher in females than in males and could therefore represent a robust means to distinguish between the sexes. A global X chromosome methylation value was calculated for each individual by summing the individual beta values at each of the 999 X chromosome CpG sites. Global X chromosome methylation across the whole cohort (n = 7,333) was approximately normally distributed (mean ± sd: 395 ± 83; kurtosis = 2.95, skewness = 0.21). For those of known sex (n = 5,207), global X chromosome methylation was significantly higher in females (mean ± sd: 455 ± 52) compared to males (mean ± sd: 328 ± 48; Welch Two Sample t-test *P* <2.2e-16).

Sex was also inferred by global X chromosome methylation values using the midpoint between the mean global X chromosome methylation values for males and females (391.5). Of the 5,147 recorded sexes (2,277 males and 2,870 females), using global X chromosome methylation to identify males gave a sensitivity (percentage of true males correctly identified) of 91.7% (2,088/2,277) and a specificity (percentage of true females correctly identified) of 89.3% (2,563/2,870) with an overall percentage of 90.7% samples where sex was correctly identified. By comparison, using PC1 to identify males gave a sensitivity (percentage of true males correctly identified) of 93.4% (2,127/2,277) and a specificity (percentage of true females correctly identified) of 93.4% (2,682/2,870) with an overall percentage of 93.4% samples in which sex was correctly identified. Given that PC1 was a more sensitive and specific method than using global X chromosome methylation, we used PC1 to re-classify all sexes in the sample (n = 7,333), resulting in a population of 3,647 females and 3,686 males.

Inspecting all samples (n = 7,333) after sex re-classification revealed some outliers, which were removed (Additional file 1: Figure S2 and S3), leaving a final population of n = 6,795, with n = 5,016 samples of recorded sex. Of these 5,016, 94.6% were consistent with sex as classified using PC1 (Additional file 2: Table S1).

**Figure 2:**
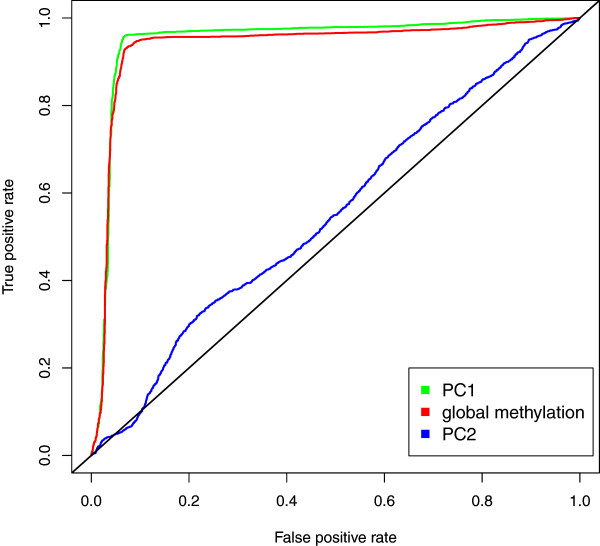
**Receiver operating characteristic (ROC) curve comparing the predictive ability of three of the metrics generated from the X chromosome methylation data; PC1, PC2 and global X chromosome methylation.**

For this final sample of n = 6,795, density plots of global X chromosome methylation by sex revealed distinct peaks using sex as assigned by PC1 (Additional file 1: Figure S3D). Global X chromosome methylation was significantly higher in females (mean ± sd: 463.9 ± 45.6) compared to males (mean ± sd: 314.9 ± 29.0; Welch Two Sample t-test *P* <2.2e-16) using sex as assigned by PC1.A receiver-operating characteristic (ROC) curve comparing the predictive ability of three metrics generated from the X chromosome methylation data (PC1, PC2 and global X chromosome methylation) showed that PC1 was the best predictor of sex (Figure 2). The area under the curve (AUC) was 0.948 for PC1 versus 0.936 for global X chromosome methylation, and only 0.553 for PC2.

### Differences in autosomal methylation between sexes

**Figure 3:**
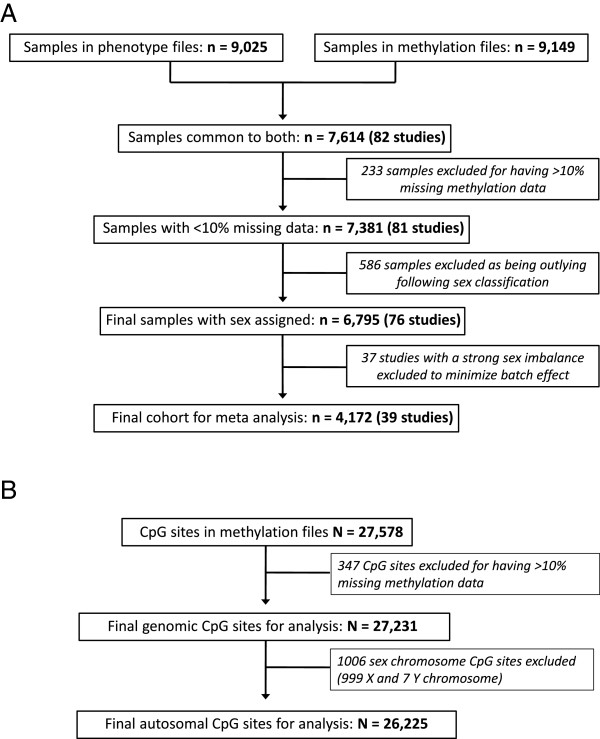
**Flow chart of sample quality control (A) and CpG quality control (B) for the data downloaded from the European Bioinformatics Institute (EBI) database.** PBL: Peripheral blood leukocyte.

**Figure 4:**
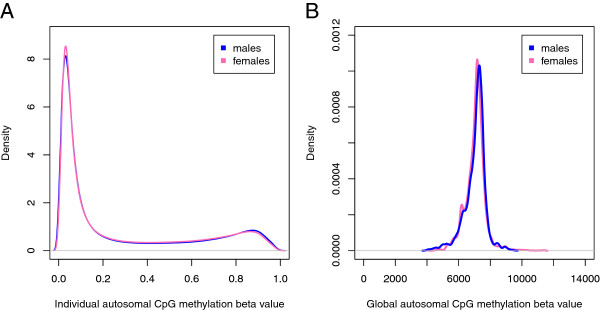
**Density plots of methylation beta values at individual CpG sites (A) and global autosomal methylation (B) across all 26,225 autosomal CpGs in all 6,795 samples, coloured by sex.**

Following quality control, 27,231 CpG sites on the HumanMethylation27K chip remained for analysis in 6,795 individuals who were successfully classified by sex (Figure 3(A) and (B)). Of these, 26,225 CpG sites were located on the autosomes (Figure 3(B)). A density plot of individual methylation beta values for each of the 26,225 autosomal CpG sites for all 6,795 individuals (Figure 4(A)) showed that across all studies and for both sexes, the majority (68%) of CpG sites had methylation values <0.3, whilst 17.4% of CpG sites had beta values >0.7, the range at which probes would be considered to be fully methylated [20, 21]. These percentages were not substantially different by sex (Additional file 2: Table S2).

Global methylation across the 22 autosomes was calculated for each sample by summing the individual CpG beta values across the 26,225 autosomal CpG sites. Global autosomal methylation was approximately normally distributed (mean ± sd: 7055 ± 652; kurtosis = 6.6, skewness = -0.31) but there were a number of female outliers (Figure 4(B) and Additional file 1: Figure S4), which skewed the mean female global autosomal methylation value (Additional file 2: Table S3). Median global autosomal methylation in males was slightly, yet statistically significantly higher than in women (median [IQR]: males 7,190 [6,770-7,426], females 7,135 [6,754-7,368]), Wilcoxon rank sum *P* =5.2e-05).

Global methylation across autosomal CpG sites varied greatly between studies (Additional file 1: Figure S5). Given that this variability could be due to underlying batch or cohort effects, a random effects meta-analysis was conducted for studies that included ≥20 individuals, and in which both sexes were represented in at least a 1:4 ratio (Additional file 3). A total of 39 studies (n = 4,172) met these criteria, of which 10 included cancer samples. The difference in global autosomal methylation according to sex in each of these studies is shown in Additional file 1: Figure S6. Meta-analysis revealed that global autosomal methylation was indeed very heterogeneous across studies, with the proportion of global autosomal variation in study estimates that is due to heterogeneity (I^2^) =87.9% [95% CI: 84.5%; 90.6%, *P* < 0.0001]. However, a funnel plot of the 39 studies did not indicate any major outliers (Additional file 1: Figure S7) and the 10 cancer studies clustered well with the 29 non-cancer studies. Despite the high heterogeneity, application of a random effects model suggested that global autosomal methylation was nominally higher in males than in females (mean beta difference = 48.9, *P* = 0.049).

**Figure 5:**
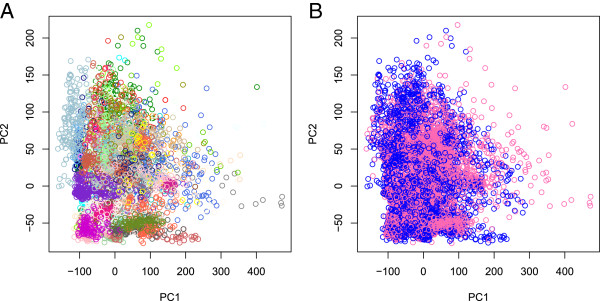
**Principal Components plots using the 1** ^**st**^ **two principal components calculated using individual beta values at all autosomal CpG sites adjusted for study, coloured by study (A) and sex (B).**

PCA of individual beta values at autosomal CpG sites by sex was also highly confounded by batch effect, even after adjustment for study (Figure 5). Hence, to identify individual CpG sites differentially methylated between males and females, we conducted a random effects meta-analysis using the 39 studies (n = 4,172) in which both sexes were represented. Estimated overall effects (mean difference in beta values between females and males, weighted by study) and corresponding *P* values were calculated. Overall, 235 (0.9%) of the 26,225 CpG sites were associated with sex after Bonferroni correction (*P* <1.9e-06).

### Autosomal gene-specific methylation

All previously identified non-specific or polymorphic CpG probes (as described by Chen and colleagues [18]) on the HumanMethylation27K array were cross-referenced against the 26,225 autosomal CpGs in the meta-analysis. In total, 2,783 of the 26,225 autosomal CpGs were non-specific and 838 were polymorphic. Of the 235 CpG sites associated with sex after Bonferroni correction in this study, 48 probes are non-specific and of the remaining 187 probes, 3 were polymorphic. These probes are indicated in the full results table (Additional file 4).

Meta-analysis of the cohort excluding cancer samples (31 studies, n = 2,900) was performed to ensure results were not due to confounding by cancer samples. There was good correlation between the *P* values from both meta-analyses, with linear regression r^2^ = 0.33, *P* < 2.2e-16 (Additional file 1: Figure S8). In addition, the majority (150/235) of the CpG sites that passed Bonferroni correction (*P* < 1.91e-06) in the meta-analysis of all samples passed the same threshold in the meta-analysis excluding cancer samples. The remaining 85 had *P* values between 1.94e-06 and 1.28e-02 in the meta-analysis excluding cancer samples (Additional file 4).

Mean differences in beta values between females and males were small. The mean beta difference between sexes for the 184 statistically significant probes was 0.037 (3.7%, Additional file 2: Table S4), with the vast majority of associated CpG sites (n = 178, 97%) more methylated in females than in males.

**Figure 6:**
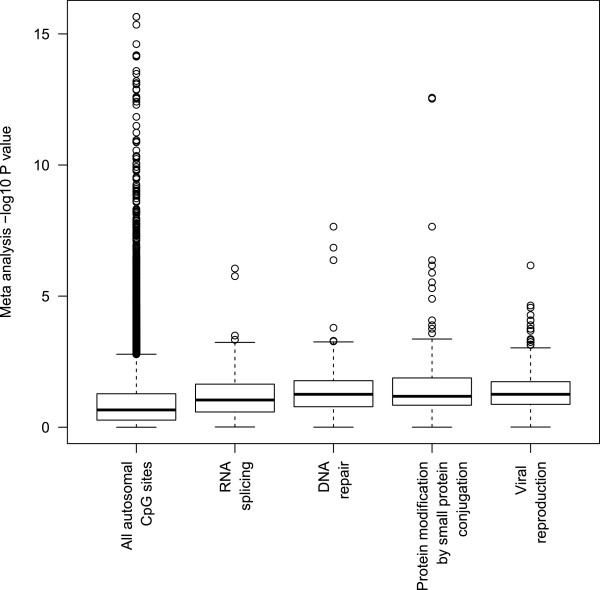
**Boxplot of meta analysis –log10** ***P*** **for all 26,** **225 autosomal CpGs, compared to those located in the genes included in the top four enriched GO Biological Processes; RNA splicing (n = 112 genes), DNA repair (n = 116 genes), protein modification by small protein conjugation (n = 122 genes) and viral reproduction (n = 140 genes).**

Pathway analysis revealed significant enrichment of genes with sex-associated changes in CpG methylation in 53 GO Biological Pathways at *P* < 0.05 (FDR adjusted). All biological processes enriched at *P* < 0.01 (FDR adjusted) are shown in Table 2 and largely comprise cellular ‘housekeeping’ functions. Gene overlap between pathways was relatively low, with 367 (73%) of the 500 genes in the top four processes (RNA splicing, DNA repair, protein modification by small protein conjugation and viral reproduction) unique to only one of these pathways (Additional file 1: Figure S9). These top four processes were relatively distinct from the individually most strongly sex-associated CpG sites, with only 10 genes from these processes represented in the CpG sites which passed Bonferroni correction. The median –log10 *P* values of the CpG sites of the genes in the top four biological processes compared to *P* values across all CpG sites are displayed in Figure 6.

**Table 2:** **All results P** < **0.01** (**FDR adjusted**) **from the NetGestalt Pathway Analysis**

GO Biological Process	GO Accession	Number of genes	***P***value (FDR adjusted)	D
RNA splicing	GO:0008380	122	5.73E-04	0.21
DNA repair	GO:0006281	116	5.73E-04	0.17
Protein modification by small protein conjugation	GO:0032446	122	5.73E-04	0.17
Viral reproduction	GO:0016032	140	5.73E-04	0.15
mRNA processing	GO:0006397	163	1.13E-03	0.18
Interphase of mitotic cell cycle	GO:0051329	170	1.13E-03	0.16
Protein catabolic process	GO:0030163	196	1.86E-03	0.15
Interphase	GO:0051325	172	1.86E-03	0.15
RNA splicing, via transesterification reactions	GO:0000375	83	2.31E-03	0.22
Protein ubiquitination	GO:0016567	119	2.76E-03	0.15
Translation	GO:0006412	101	3.53E-03	0.16
rRNA metabolic process	GO:0016072	30	7.11E-03	0.33
Chromatin modification	GO:0016568	97	7.11E-03	0.15
M phase	GO:0000279	165	7.84E-03	0.13
Cellular protein catabolic process	GO:0044257	164	8.92E-03	0.14
rRNA processing	GO:0006364	28	9.76E-03	0.33

GO: Gene Ontology. D: D-statistic; the maximum difference in cumulative fraction (of *P* value distribution). Only positive associations are shown (pathways enriched for genes containing CpG sites with lower *P* values compared to all CpG sites). The number of genes in each pathway that were represented by CpG sites in the meta-analysis is also shown.

## Discussion

In this study we conducted a meta-analysis of publicly available, genome-wide methylation datasets to examine the possible relationship between methylation profiles and sex. Following rigorous quality control, we analysed the data for differential methylation patterns in terms of global X chromosome methylation, global autosomal methylation, and differences between the methylation status at individual autosomal CpG probes according to sex.

### X chromosome analysis

The X chromosome analysis demonstrated that, as expected, global X chromosome methylation is considerably higher in females compared to males. We also demonstrated that it is possible to accurately infer sex, based on PCA of X chromosome methylation data, and that this method is superior to using global X chromosome methylation. In light of the increasing evidence for the importance of correction for sex in analysis of disease-specific methylation patterns, this provides a valuable technique for studies where sex information is not easily accessible.

### Autosomal methylation analysis

This analysis showed a small but significant increase in global autosomal methylation associated with male sex, concurring with previous studies [7]. Many mechanisms may account for this global difference, but have so far remained elusive. A recent study indicated that this global difference in autosomal methylation does not appear to be driven by sex hormones [22].

The findings of the analysis of methylation at individual CpG sites in this study have a number of features in common with the findings of Chen *et al*. [18]. After excluding probes which were found to be non-specific, Chen *et al*. [18] also found only small absolute differences between male and female samples in the CpG probes that they found to be significantly differentially methylated by sex (2-12%). Similar to this study, the majority (5/7) of the CpG sites that they found to be associated with sex were more methylated in females. Chen and colleagues reported associations between three (cg08124399 in *DDX43*, cg08532057 in *NUPL1* and cg18485485 in *DECR1*) of our top 20 CpG sites and similar differences in beta values between sexes [18].

As predicted, the majority of the genes that Liu *et al*. [13] found to be differentially methylated according to sex were on the X chromosome. These investigators reported 11 genes with very small CpG differences in autosomes: *LRRC2 TDGF1*, *RAB9P1*, *C6ORF68*, *TLE1*, *GLUD1*, *ALX4*, *DPPA3*, *NUPL1*, *FLJ20582* and *FLJ43276*. All of these genes were found to be significant in our analysis (Additional file 3), with most being among our top 20 loci. Apart from the *NUPL1* CpG probe, however, all of these CpG sites were found to be non-specific by Chen *et al*. [18].

Autosomal sex-specific DNA methylation has been demonstrated in several candidate gene studies [5, 6, 14, 23, 24]. Although some of these genes (*CDKN2A*, *MTHFR* and *MGMT*) are also interrogated to some extent by the HumanMethylation27K array, we observed significant sex-specific DNA methylation only with *MGMT* (Additional file 3), with all *MGMT* probes being more methylated in females. These findings are in contrast to previous reports in which higher methylation was shown in males [5, 24].

Genes containing CpG islands with methylation differences of >5% between the sexes are listed in Table 3. Many of these top CpG associations are in genes that may play a role in sex-specific functions. The top-hit from our meta-analysis, *SLC9A2*, encodes Solute Carrier Family 9. Other Solute Carrier Family proteins have been shown to be associated with male infertility [25], suggesting a possible sex-specific role for this class of proteins. *DDX43* has been shown to be differentially expressed in normal testis compared to testis of patients diagnosed with *Sertolicell*-*only* (*SCO*) syndrome, and may play a role in spermatogenesis [25]. *NUPL1* has been shown to be highly expressed in the testes of fertile men compared to that of infertile men, suggesting its function is important for male fertility [25]. These genes may be associated with male infertility secondary to epigenetic deregulation. The results of the pathway analysis, on the other hand, suggest that the influence of sex on methylation is broad. We report enriched biological processes across a wide range of cellular functions, some of which, including DNA transcription and RNA splicing, were also reported by Liu *et al*. [13].

**Table 3:** **CpG sites with a differences in methylation of** >**5**% **between males and females in meta**-**analysis of 4**,**172 samples**

GENE	CHR	Target ID	Meta-analysis ***P***value	Meta-analysis Δ beta	NCBI description of protein function	n studies consistent effect
*SLC9A2*	2	cg20050113	<2.2e-16	-0.09	Involved in pH regulation to eliminate acids generated by active metabolism or to counter adverse environmental conditions.	36
*DDX43*	6	cg08124399	<2.2e-16	-0.07	ATP-dependent RNA helicase in the DEAD-box family.	39
*SPESP1*	15	cg09886641	<2.2e-16	-0.06	Human alloantigen involved in sperm-egg binding and fusion.	37
*FIGNL1*	7	cg05072008	<2.2e-16	-0.06	May regulate osteoblast proliferation and differentiation.	36
*CRISP2*	6	cg04595372	<2.2e-16	-0.06	Also known as Testis-Specific Protein TPX-1. May regulate some ion channels’ activity and thereby regulate calcium fluxes during sperm capacitation.	36
*NUPL1*	13	cg08532057	<2.2e-16	-0.06	Component of the nuclear pore complex, a complex required for the trafficking across the nuclear membrane.	30

Direction of effect (Δ beta) is for male relative to female methylation beta value, so that negative values indicate lower methylation in males. The number of the 39 studies in the meta-analysis which had individual associations consistent with the direction of effect in the meta analysis are also shown. All listed probes are located within a CpG island.

Since the completion of this study, an increasing amount of data from the HumanMethylation450K microarray are becoming publicly available, along with tools for their analyses [26]. The 450K array offers higher-resolution methylation data than the 27K, and it will be interesting see what this new set of data will reveal about sex-specific methylation patterns.

## Conclusions

With the inclusion and careful analysis of all publicly available datasets, this study represents the most comprehensive analysis of sex-specific methylation patterns to date. This is likely the reason that, unlike previous studies, which reported few detectable DNA methylation differences in autosomal genes between sexes [13, 18], we identified a modest number of CpG sites associated with sex after Bonferroni correction. Similar to other studies using microarray platforms, however, the mean beta differences associated with sex are very small (approximately 5%) [13, 14, 18]. This is far below the smallest difference (approximately 17%) that microarray platforms reliably estimate [27]. The reason for this is not clear; it may be that sex-associated methylation differences are highly dynamic, leading to heterogeneous samples. Nonetheless, the results reported here reliably demonstrate the existence of sex-specific methylation profiles, which is important not only in a biological context, but in recognising and dealing with potential confounding when undertaking methylation-disease association studies.

## Methods

### Data collation and quality control

We extracted publicly available methylation datasets from the European Bioinformatics Institute (EBI) database (http://www.ebi.ac.uk/arrayexpress). The vast majority of studies in this repository were conducted using the Illumina Infinium HumanMethylation27K microarray to assess genome-wide DNA methylation, and we therefore chose to analyse studies using this platform. This microarray uses 50-mer oligonucleotide probes to target 27,578 CpG sites covering ~14,000 autosomal and sex chromosome genes. These CpGs map to the promoter regions of genes with an average coverage of two CpGs per gene and more extensive coverage (3–20 CpGs) for cancer-related and imprinted genes. Infinium technology has previously been described for SNP genotyping [28]. In order to detect methylation differences, DNA is treated with sodium bisulfite, which converts unmethylated cytosines to uracil, whereas methylated cytosine is protected and remains unchanged. Two probes are designed for each CpG site—one is specific for the methylated allele (cytosine) and the other for the unmethylated allele (thymidine). The DNA methylation level for a CpG site is determined by dividing the signal intensity for the methylated CpG by the sum of both the methylated and unmethylated CpGs, previously shown to be a reliable estimate of the level of methylation at a locus [20]. This is referred to as the ‘beta value’ and approximates to the percent methylation divided by 100. Previous studies have shown that probes with beta values <0.3 represent unmethylated areas of the genome [20] and we therefore defined unmethylated probes on this basis. In cell lines, probes with beta >0.7 represent genomic loci that are fully methylated; however, as tissue samples frequently comprise a mixture of cell types and therefore a mixture of methylated and unmethylated probes, we set a beta value threshold of >0.3 to define methylated probes [20, 21]. In this study, in addition to analysing beta values at individual CpG sites, we also investigated global methylation values. A global methylation value was calculated for each individual in the study as the sum of the beta values at each CpG site, either across the X chromosome (global X chromosome methylation), or across all autosomes (global autosomal methylation).

Experiments from the EMBL-EBI database (http://www.ebi.ac.uk/arrayexpress) using the Illumina HumanMethylation27K BeadChip (HumanMethylation27_270596_v.1.2) up to March 2013 were incorporated into the study. As the majority of experiments made processed but not raw methylation data available in the EMBL-EBI database, we included only processed (normalised) datasets. Initially, 92 studies were retrieved from the EBI database. Samples not of human origin, and all samples derived from cell lines were excluded from further analysis. Duplicate entries were removed. Datasets that appeared to have been incorrectly processed (for example, those containing beta values <0 or >1) were excluded. Datasets which reported non- standard CpG site identifiers were also excluded.

Following dataset quality control, phenotype and methylation files were merged. There were 7,614 samples from 84 studies for whom matching methylation and phenotype data were available (Figure 3A). A list of the EBI Accession numbers for these studies, and the number of samples included from each study, is provided in Additional file 5.

Within each study, probes with reported detection *P* values (generated by Illumina GenomeStudio Software as an objective measure of overall probe performance) >0.05 were removed from the analysis. In the merged dataset of 7,614 samples, all CpG sites and samples with >10% missing methylation data were excluded. Consequently, the final dataset comprised 27,231 CpG sites from 7,381 samples (Figure 3A and B).

Following the exclusion of CpG sites and samples with >10% missing methylation data, 0.13% of the remaining 27,231 CpG sites from 7,381 samples had missing beta values. The median number of missing beta values per CpG site was 3 (IQR 1-5, range 0-191). The median number of missing beta values per sample was 8 (IQR 3-15, range 0-298). Missingness was at random (the probability of missingness was not significantly related to any observed variables – sex, study, CpG site nor individual).

Given that missingness rates were low, and randomly distributed, we did not anticipate missingness to be a problem in this analysis. Missing beta values were removed from the analysis of individual CpG probes.

### Sex assignation using X chromosome data

The HumanMethylation27K Chip contains 1,085 CpG probes on the X chromosome. Of these, 86 were removed during quality control (Figure 3(B)), leaving 999 X chromosome CpG sites for analysis. A principal components analysis (PCA) was performed in R, using the prcomp function in the R package ‘stats’ [29]. One study (accession ID: E-GEOD-23311, ‘DNA Methylation in Human Chorionic Villus and Maternal Blood Cells’) was excluded due to being a major outlier on the PCA (Additional file 1: Figure S1).

### Meta-analysis of individual CpG probes

A meta-analysis was performed to investigate associations between individual CpG probes and sex in a subset of studies that included at least 20 individuals, and both sexes in a ratio of at least 1:4 (Additional file 4). The meta-analysis was carried out using the R package ‘meta’ [29]. Inverse variance weighting was used for pooling. The DerSimonian-Laird estimate for the between-study variance was used in a random effects model [30]. To ensure that the meta-analysis results were not confounded by cancer samples, a second meta-analysis was performed using the same protocol. All 1,240 cancer samples from the original 4,172 were excluded from this analysis, leaving 31 studies (n = 2,900) that had > 20 individuals and were composed of both sexes.

### Pathway analysis of the associations between individual CpG probes and sex

We conducted a pathway analysis using –log10 *P* values of all CpG probes from the meta-analysis, annotated by gene name. The NetGestalt web application was used to integrate these continuous data over GO biological processes using random walk distance-based hierarchical clustering for module identification [31]. Enriched modules were identified using the Kolmogorov-Smirnov test [32, 33]. The Benjamini–Hochberg method was used for controlling the false discovery rate (FDR) [34]. All annotated genes on the Infinium HumanMethylation27 arrays were used as the background list against which enriched-GO terms in target lists of genes were compared.

## Electronic supplementary material

Additional file 1: Figure S1: The 1^st^ two PCs from the 999 X chromosome CpG sites in the initial 82 studies (n = 7,381). **Figure S2.** Distribution of PC1 and global methylation values across all 999 X chromosome sites in all samples (n = 7,333) and samples with sex recorded only (n = 5,147) coloured by sex. **Figure S3.** Distribution of global methylation values across all 999 X chromosome sites **(A)** and PC1 from X chromosome CpG sites **(B)** by sex after exclusion of spurious ‘male’ samples (n = 6,812), PC plot of X chromosome showing further outliers **(C)** and density plot of final dataset (n = 6,795) **(D)**. **Figure S4.** Boxplot of female and male global autosomal methylation (n = 6,795). **Figure S5.** Density of global autosomal CpG methylation in the final cohort (n = 6,795), coloured by study. **Figure S6.** Boxplots of global autosomal methylation by sex in each study in the meta-analysis (n = **Figure S7.** Funnel plot for meta-analysis of global autosomal methylation by sex (n = 4,172, 39 studies). **Figure S8.** Meta-analysis *P* values for all samples (n = 4,172, 39 studies) plotted against those for the meta-analysis excluding cancer samples (n = 2,900, 31 studies). **Figure S9.** Venn diagram of the top four GO biological processes which were enriched for genes differentially methylated by sex in the pathway analysis. (DOCX 2 MB)

Additional file 2: Table S1: Comparison of the sex assigned by recorded phenotype, PC1 and global X chromosome methylation in the final dataset (n = 6,795). **Table S2.** Percentage of the 26,225 autosomal CpGs in each methylation category according to sex. **Table S3.** Summary of the global methylation across the 24,225 autosomal CpG sites (n = 6,795 samples). **Table S4** Average absolute difference in beta value by sex in CpG methylation across all 184 non cross-reactive, non-polymorphic CpG sites which passed Bonferroni Correction. (DOCX 20 KB)

Additional file 3: **All studies from the original cohort (76 studies, n = 6,795) which included ≥20 individuals and were comprised of both sexes in a ratio of at least 1:4 were included in the meta analysis.** (XLSX 18 KB)

Additional file 4: **The 235 autosomal CpG sites associated with sex in the meta-analysis (n = 4,172, 39 studies), after Bonferroni Correction for multiple testing (** ***P*** **<1.918e-06).** All three of the probes with beta differences of >10% in the meta-analysis [cg15915418 in TLE1 (Δ beta = 31%); cg27063525 in C6orf69 (Δ beta = 17%) and cg11673803 in GLUD1 (Δ beta = 15%)] were non-specific, and feature highly in the top non-specific probes reported by Chen et al. All three of these 50-mer probes have 100% match identity to non-target sequences on the X chromosome. Of the 235 CpG sites which were significant after Bonferroni correction, 31 of the top 100 CpG probes by beta difference were cross reactive. (XLSX 43 KB)

Additional file 5: **A list of the EBI Accession numbers for the initial dataset of 7,614 samples from 84 studies, and the number of samples included from each study.** (XLSX 11 KB)

## Abbreviations

GO: Gene ontology
PCA: Principal component analysis
PC: Principal component
ROC: Receiver-operating characteristic
AUC: Area under the curve.

## References

[1] Jones PA, Takai D (2001). The role of DNA methylation in mammalian epigenetics. Science.

[2] Jones PA (2012). Functions of DNA methylation: islands, start sites, gene bodies and beyond. Nat Rev Genet.

[3] Boyes J, Bird A (1991). DNA methylation inhibits transcription indirectly via a methyl-Cpg binding-protein. Cell.

[4] Robertson KD (2005). DNA methylation and human disease. Nat Rev Genet.

[5] Sarter B, Long TI, Tsong WH, Koh WP, Yu MC, Laird PW (2005). Sex differential in methylation patterns of selected genes in Singapore Chinese. Hum Genet.

[6] El-Maarri O, Becker T, Junen J, Manzoor SS, Diaz-Lacava A, Schwaab R, Wienker T, Oldenburg J (2007). Gender specific differences in levels of DNA methylation at selected loci from human total blood: a tendency toward higher methylation levels in males. Hum Genet.

[7] El-Maarri O, Walier M, Behne F, Van Uum J, Singer H, Diaz-Lacava A, Nusgen N, Niemann B, Watzka M, Reinsberg J, van der Ven H, Wienker T, Stoffel-Wagner B, Schwaab R, Oldenburg J (2011). Methylation at global LINE-1 repeats in human blood are affected by gender but not by age or natural hormone cycles. PLoS One.

[8] Zhang FF, Cardarelli R, Carroll J, Fulda KG, Kaur M, Gonzalez K, Vishwanatha JK, Santella RM, Morabia A (2011). Significant differences in global genomic DNA methylation by gender and race/ethnicity in peripheral blood. Epigenetics.

[9] Burghardt KJ, Pilsner JR, Bly MJ, Ellingrod VL (2012). DNA methylation in schizophrenia subjects: gender and MTHFR 677C/T genotype differences. Epigenomics.

[10] Tapp HS, Commane DM, Bradburn DM, Arasaradnam R, Mathers JC, Johnson IT, Belshaw NJ (2013). Nutritional factors and gender influence age-related DNA methylation in the human rectal mucosa. Aging Cell.

[11] Subramanyam MA, Diez-Roux AV, Pilsner JR, Villamor E, Donohue KM, Liu Y, Jenny NS (2013). Social factors and leukocyte DNA methylation of repetitive sequences: the multi-ethnic study of atherosclerosis. PLoS One.

[12] Fuke C, Shimabukuro M, Petronis A, Sugimoto J, Oda T, Miura K, Miyazaki T, Ogura C, Okazaki Y, Jinno Y (2004). Age related changes in 5-methylcytosine content in human peripheral leukocytes and placentas: an HPLC-based study. Ann Hum Genet.

[13] Liu J, Morgan M, Hutchison K, Calhoun VD (2010). A study of the influence of sex on genome wide methylation. PLoS One.

[14] Boks MP, Derks EM, Weisenberger DJ, Strengman E, Janson E, Sommer IE, Kahn RS, Ophoff RA (2009). The relationship of DNA methylation with age, gender and genotype in twins and healthy controls. PLoS One.

[15] Adkins RM, Krushkal J, Tylavsky FA, Thomas F (2011). Racial differences in gene-specific DNA methylation levels are present at birth. Birth Defects Res A Clin Mol Teratol.

[16] Adkins RM, Thomas F, Tylavsky FA, Krushkal J (2011). Parental ages and levels of DNA methylation in the newborn are correlated. BMC Med Genet.

[17] Eckhardt F, Lewin J, Cortese R, Rakyan VK, Attwood J, Burger M, Burton J, Cox TV, Davies R, Down TA, Haefliger C, Horton R, Howe K, Jackson DK, Kunde J, Koenig C, Liddle J, Niblett D, Otto T, Pettett R, Seemann S, Thompson C, West T, Rogers J, Olek A, Berlin K, Beck S (2006). DNA methylation profiling of human chromosomes 6, 20 and 22. Nat Genet.

[18] Chen YA, Choufani S, Ferreira JC, Grafodatskaya D, Butcher DT, Weksberg R (2011). Sequence overlap between autosomal and sex-linked probes on the Illumina HumanMethylation27 microarray. Genomics.

[19] Chen YA, Choufani S, Grafodatskaya D, Butcher DT, Ferreira JC, Weksberg R (2012). Cross-reactive DNA microarray probes lead to false discovery of autosomal sex-associated DNA methylation. Am J Hum Genet.

[20] Sproul D, Nestor C, Culley J, Dickson JH, Dixon JM, Harrison DJ, Meehan RR, Sims AH, Ramsahoye BH (2011). Transcriptionally repressed genes become aberrantly methylated and distinguish tumors of different lineages in breast cancer. Proc Natl Acad Sci U S A.

[21] Sproul D, Kitchen RR, Nestor CE, Dixon JM, Sims AH, Harrison DJ, Ramsahoye BH, Meehan RR (2012). Tissue of origin determines cancer-associated CpG island promoter hypermethylation patterns. Genome Biol.

[22] Iwasaki M, Ono H, Kuchiba A, Kasuga Y, Yokoyama S, Onuma H, Nishimura H, Kusama R, Yoshida T, Tsugane S (2012). Association of postmenopausal endogenous sex hormones with global methylation level of leukocyte DNA among Japanese women. BMC Cancer.

[23] Wiencke JK, Zheng S, Lafuente A, Lafuente MJ, Grudzen C, Wrensch MR, Miike R, Ballesta A, Trias M (1999). Aberrant methylation of p16INK4a in anatomic and gender-specific subtypes of sporadic colorectal cancer. Cancer Epidemiol Biomarkers Prev.

[24] Wu JY, Wang J, Lai JC, Cheng YW, Yeh KT, Wu TC, Chen CY, Lee H (2008). Association of O6-methylguanine-DNA methyltransferase (MGMT) promoter methylation with p53 mutation occurrence in non-small cell lung cancer with different histology, gender, and smoking status. Ann Surg Oncol.

[25] Fox MS, Ares VX, Turek PJ, Haqq C, Reijo Pera RA (2003). Feasibility of global gene expression analysis in testicular biopsies from infertile men. Mol Reprod Dev.

[26] Lowe R, Rakyan V (2013). Marmal-aid - a database for infinium human methylation450. BMC Bioinformatics.

[27] Bibikova M, Lin ZW, Zhou LX, Chudin E, Garcia EW, Wu B, Doucet D, Thomas NJ, Wang YH, Vollmer E, Goldmann T, Seifart C, Jiang W, Barker DL, Chee MS, Floros J, Fan JB (2006). High-throughput DNA methylation profiling using universal bead arrays. Genome Res.

[28] Steemers FJ, Chang W, Lee G, Barker DL, Shen R, Gunderson KL (2006). Whole-genome genotyping with the single-base extension assay. Nat Methods.

[29] Development Core Team, R (2005). A Language and Environment for Statistical Computing.

[30] DerSimonian R, Laird N (1986). Meta-analysis in clinical trials. Control Clin Trials.

[31] Shi Z, Wang J, Zhang B (2013). NetGestalt: integrating multidimensional omics data over biological networks. Nat Methods.

[32] Kolmogorov A (1933). Sulla Determinazione Empirica di una Legge di Duistributione. Giornale dell’ Istituto Ialiano delgli Attuar.

[33] Smirnov N (1939). On the estimation of the discrepancy between empirical curves of distribution for two independent samples. Bull Math Univ Moscow.

[34] Benjamini Y, Hochberg Y (1995). Controlling the false discovery rate - a practical and powerful approach to multiple testing. J R Stat Soc B Met.

