# Supplementary material for: Meta-analysis of human methylation data for evidence of sex-specific autosomal patterns
Source: BMC Genomics. 2014 Nov 18;15(1):981. doi: 10.1186/1471-2164-15-981 (PMC4255932; doi:10.1186/1471-2164-15-981)
Supplement: Supplementary file 1 — Additional file 1: Figure S1: The 1st two PCs from the 999 X chromosome CpG sites in the initial 82 studies (n = 7,381). Figure S2. Distribution of PC1 and global methylation values across all 999 X chromosome sites in all samples (n = 7,333) and samples with sex recorded only (n = 5,147) coloured by sex. Figure S3. Distribution of global methylation values across all 999 X chromosome sites (A) and PC1 from X chromosome CpG sites (B) by sex after exclusion of spurious ‘male’ samples (n = 6,812), PC plot of X chromosome showing further outliers (C) and density plot of final dataset (n = 6,795) (D). Figure S4. Boxplot of female and male global autosomal methylation (n = 6,795). Figure S5. Density of global autosomal CpG methylation in the final cohort (n = 6,795), coloured by study. Figure S6. Boxplots of global autosomal methylation by sex in each study in the meta-analysis (n = Figure S7. Funnel plot for meta-analysis of global autosomal methylation by sex (n = 4,172, 39 studies). Figure S8. Meta-analysis P values for all samples (n = 4,172, 39 studies) plotted against those for the meta-analysis excluding cancer samples (n = 2,900, 31 studies). Figure S9. Venn diagram of the top four GO biological processes which were enriched for genes differentially methylated by sex in the pathway analysis. (DOCX 2 MB) [file 12864_2014_6710_MOESM1_ESM.docx]

**Figures S1 – S9.**


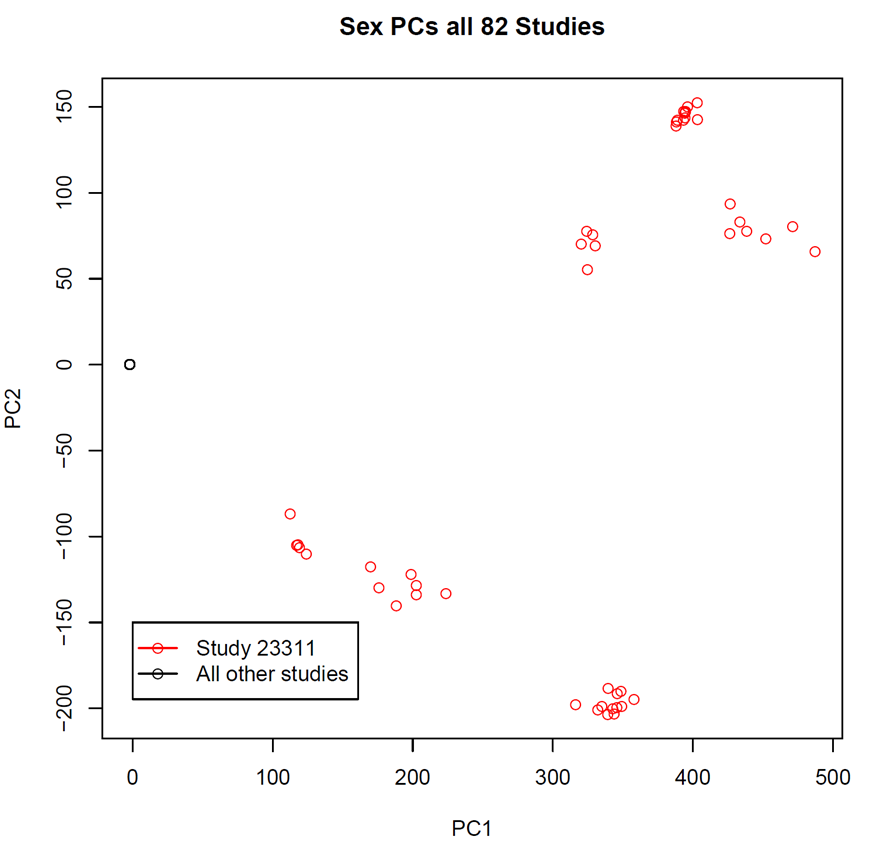


Figure S1. The 1^st^ two PCs from the 999 X chromosome CpG sites in the initial 82 studies (n=7,381). This plot shows that while samples from most studies cluster around the 0 point on the PC plot, samples from study 23311 are clear outliers. This study was therefore removed from further analyses, leaving n=7,333 samples.


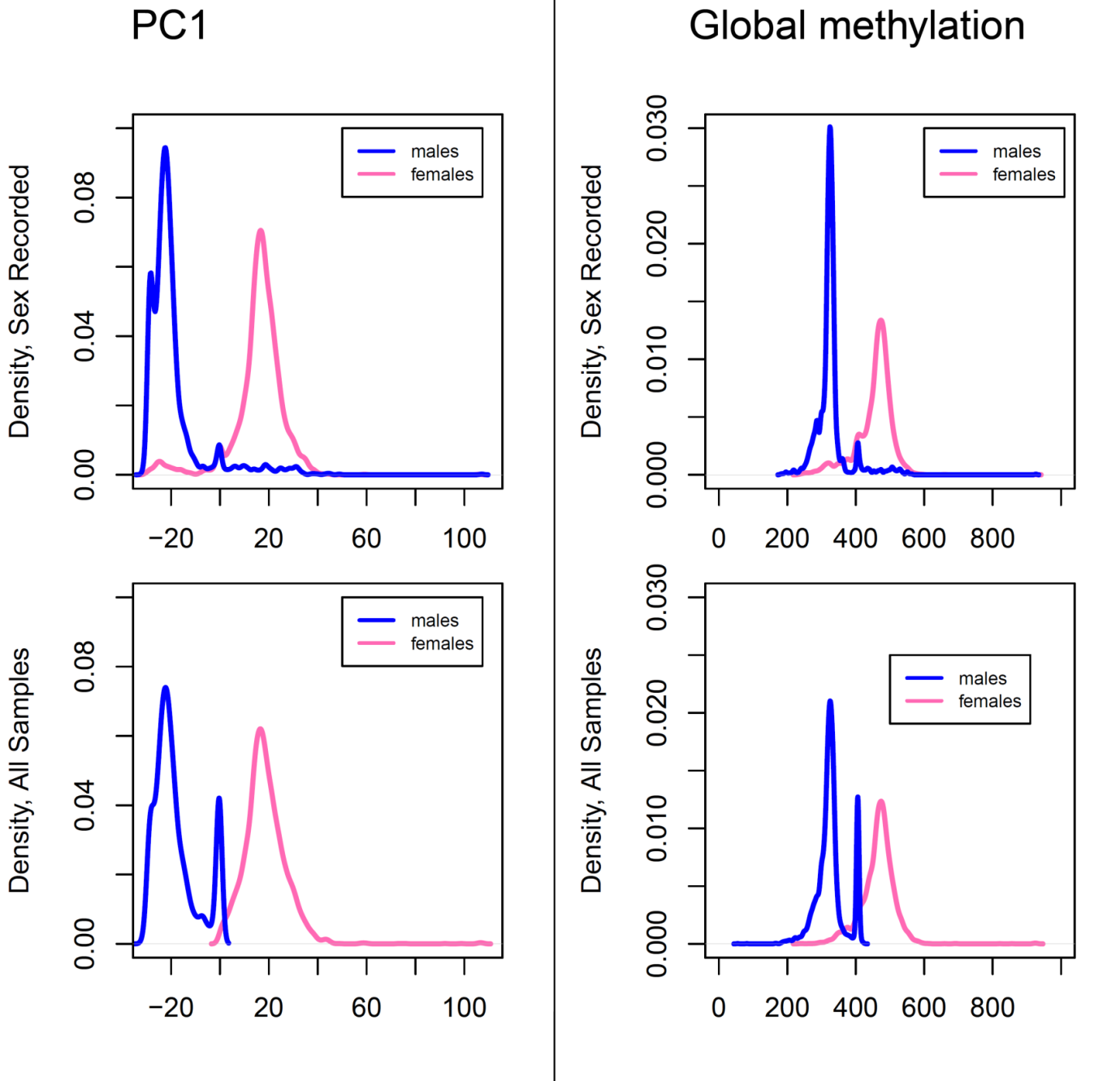


Figure S2. Distribution of PC1 and global methylation values across all 999 X chromosome sites in all samples (n=7,333) and samples with sex recorded only (n=5,147) coloured by sex. Sex was coloured according to that assigned by PC1 (3,686 males and 3,647 females). Based on this density plot, 521 spurious ‘male’ samples, which formed an independent peak close to the female distribution, were excluded using the cut-off of PC1>-5, after which 6,812 samples remained.

**Figure S3.** **Distribution of global methylation values across all 999 X chromosome sites (A) and PC1 from X chromosome CpG sites (B) by sex after exclusion of spurious ‘male’ samples (n=6,812), PC plot of X chromosome showing further outliers (C) and density plot of final dataset (n=6,795) (D).** Sex was coloured according to that assigned by PC1 (3,165 men and 3,647 women). Plots A and B indicate that there is much better separation of the male and female peaks than in following the exclusion of the 521 spurious ‘male’ samples. Plotting the 1^st^ 2 PCs (C) shows a number of outliers remain. Twelve outliers were removed based on PC1>50, and six based on PC2>60, leaving 6,795 samples for analysis (D).


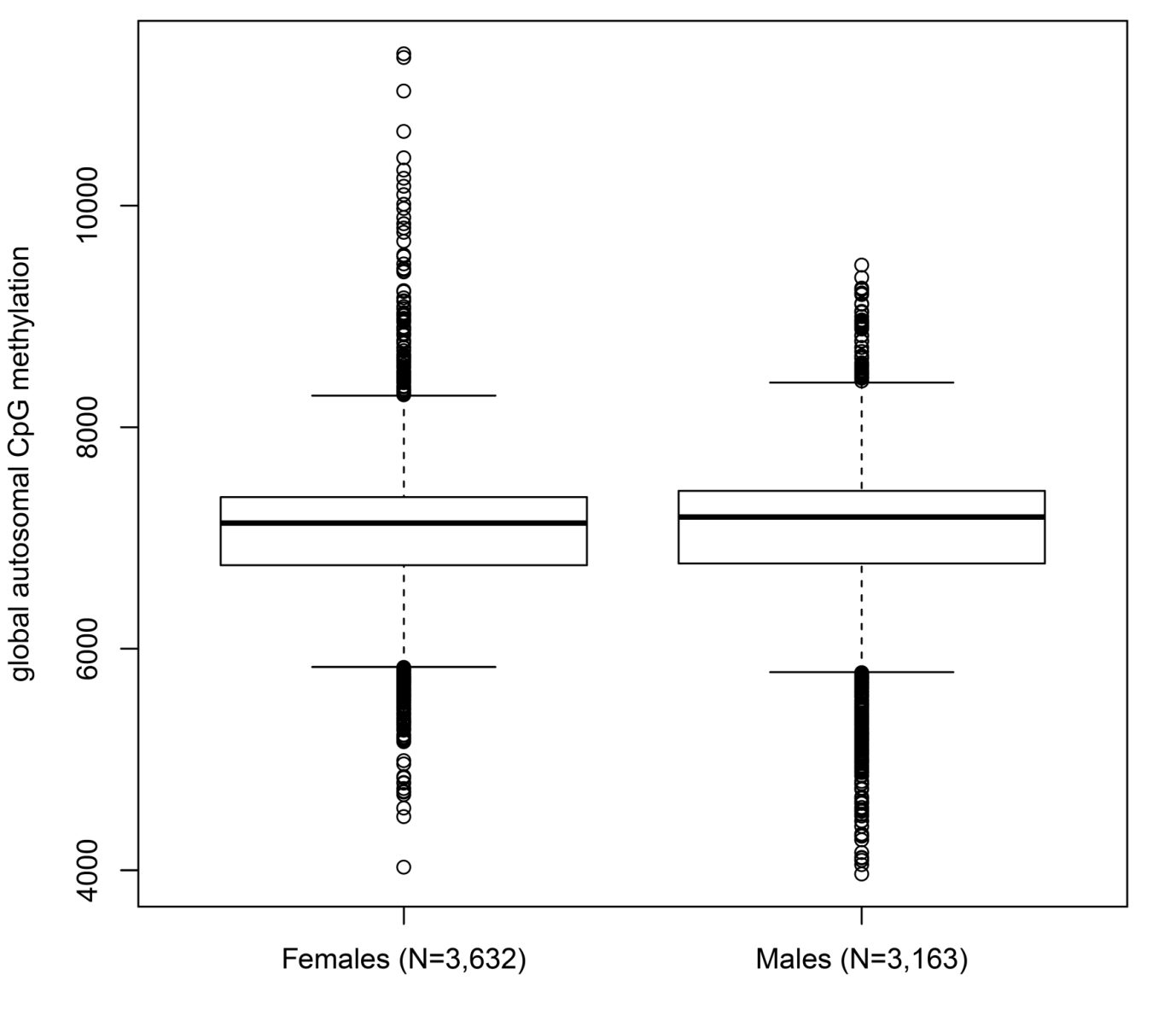


Figure S4. Boxplot of female and male global autosomal methylation (n=6,795).


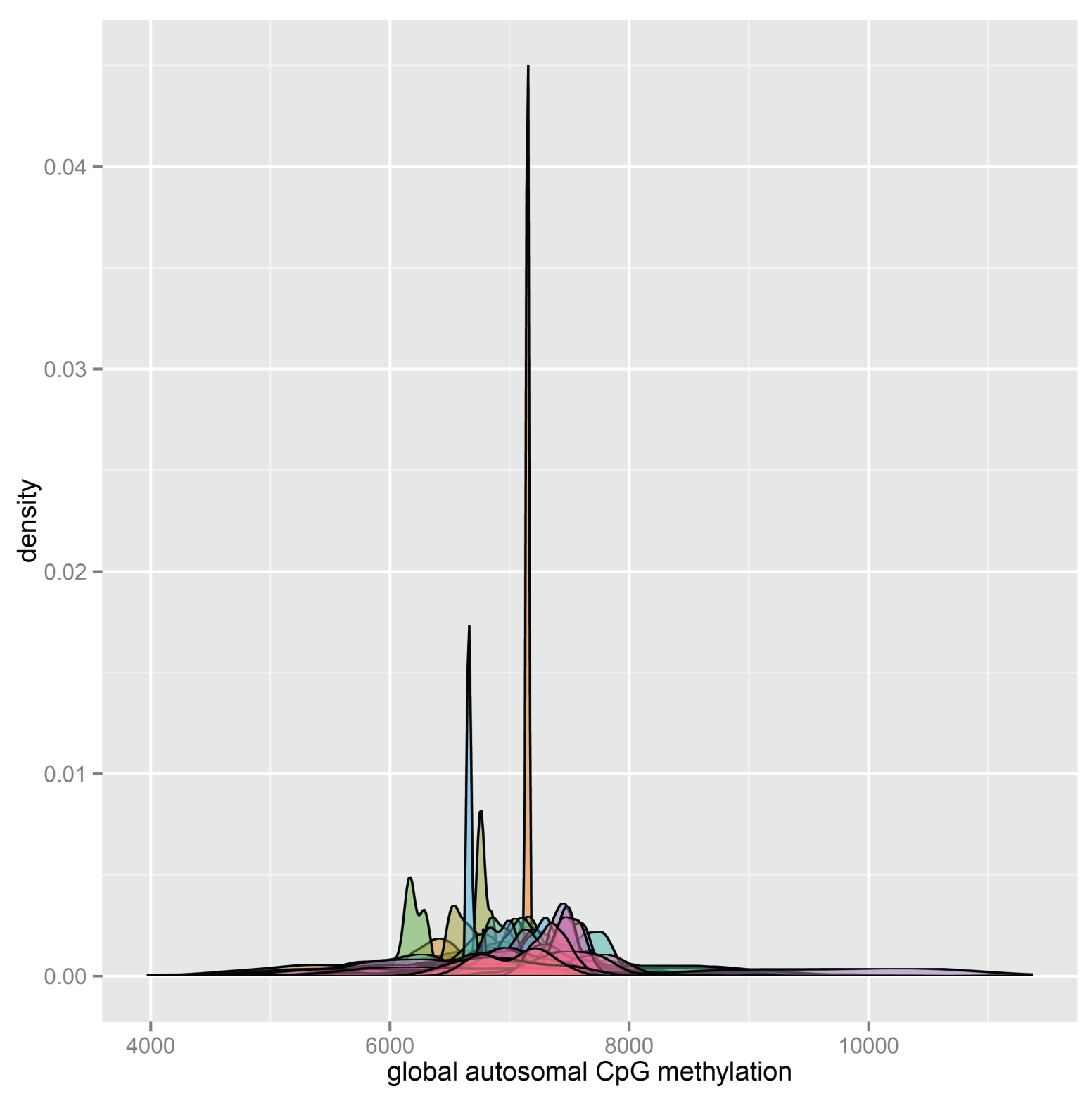


Figure S5. Density of global autosomal CpG methylation in the final cohort (n=6,795), coloured by study.


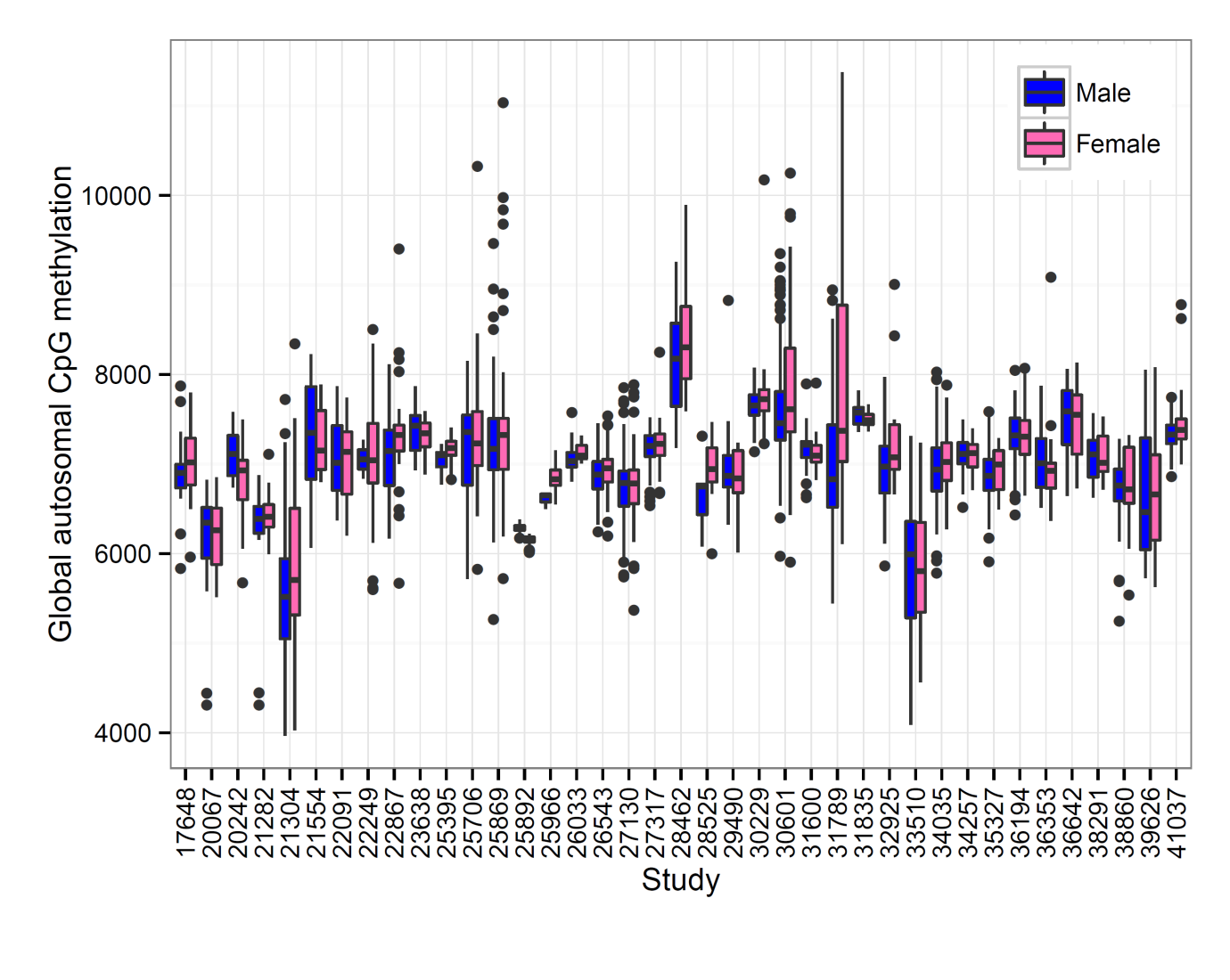


Figure S6. Boxplots of global autosomal methylation by sex in each study in the meta-analysis (n= 39 studies). Black dots represent outliers.

Figure S7. Funnel plot for meta-analysis of global autosomal methylation by sex (N=4,172, 39 studies). Studies including cancer samples are coloured in red.


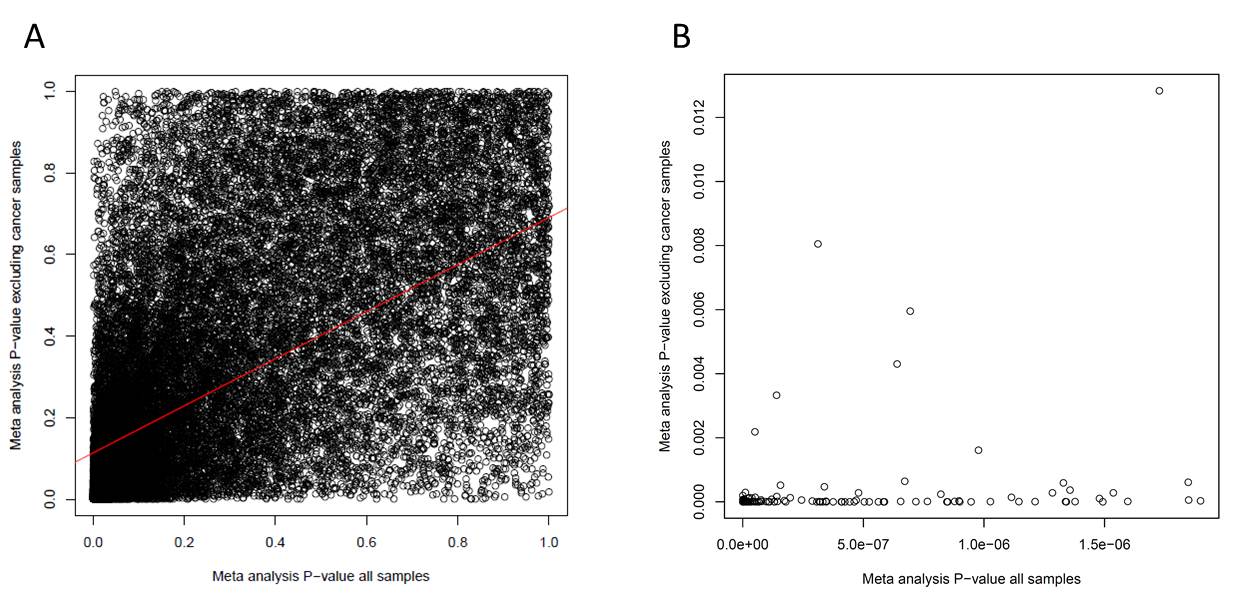


**Figure S8.** **Meta-analysis *P* values for all samples (n= 4,172, 39 studies) plotted against those for the meta-analysis excluding cancer samples (n=2,900, 31 studies).** A: All *P* values. B: *P* values for CpG sites which passed Bonferroni correction in the all-samples analysis. After exclusion of all 1,240 cancer samples from the original 4,172, 31 studies (n=2,900) remained which had>20 individuals and were comprised of both sexes. Plot A shows that there is good correlation between the P values, with linear regression r^2^=0.33, *P* value: < 2.2e-16. Plot B shows that the majority (150/235) of the CpG sites that passed Bonferroni correction (*P*<1.91e-06) in the meta-analysis of all samples passed the same threshold in the meta-analysis excluding cancer samples. The remaining (85 had *P* values between 1.94e-06 and 1.28e-02 in the meta-analysis excluding cancer samples.

Figure S9. Venn diagram of the top four GO biological processes which were enriched for genes differentially methylated by sex in the pathway analysis. These four processes were RNA splicing (RNA), DNA repair (DNA), protein modification by small protein conjugation (protein) and viral reproduction (viral). The number of genes represented by at least one CpG site in the methylation analysis in each pathway are also shown.
