# Supplementary material for: Meta-analysis of human methylation data for evidence of sex-specific autosomal patterns
Source: BMC Genomics. 2014 Nov 18;15(1):981. doi: 10.1186/1471-2164-15-981 (PMC4255932; doi:10.1186/1471-2164-15-981)
Supplement: Supplementary file 2 — Additional file 2: Table S1: Comparison of the sex assigned by recorded phenotype, PC1 and global X chromosome methylation in the final dataset (n = 6,795). Table S2. Percentage of the 26,225 autosomal CpGs in each methylation category according to sex. Table S3. Summary of the global methylation across the 24,225 autosomal CpG sites (n = 6,795 samples). Table S4 Average absolute difference in beta value by sex in CpG methylation across all 184 non cross-reactive, non-polymorphic CpG sites which passed Bonferroni Correction. (DOCX 20 KB) [file 12864_2014_6710_MOESM2_ESM.docx]

**Tables S1 – S4.**

Table S1. Comparison of the sex assigned by recorded phenotype, PC1 and global X chromosome methylation in the final dataset (n=6,795).

| **After exclusion of outliers (n=6,795)** | **Female** | | **Male** | | **Total** | |
| --- | --- | --- | --- | --- | --- | --- |
|  | **n** | **%** | **n** | **%** | **n** | **%** |
| total samples with sex recorded | 2797 | 55.8 | 2219 | 44.2 | 5016 | NA |
| sex correctly identified using PC >0 | 2677 | 95.7 | 2070 | 93.3 | 4747 | 94.6 |
| sex correctly identified using global methylation (using the midpoint of the means, 391.5) | 2510 | 89.7 | 2079 | 93.7 | 4589 | 91.5 |

Table S2. Percentage of the 26,225 autosomal CpGs in each methylation category according to sex.

|  | **n samples** | **Total CpGs*** | **% CpGs beta < 0.3** | **% CpGs 0.3 ≤ beta ≤ 0.7** | **% CpGs beta > 0.7** |
| --- | --- | --- | --- | --- | --- |
| **All samples** | 6,795 | 178,198,875 | 68.0% | 14.6% | 17.4% |
| **Male samples** | 3,163 | 82,949,675 | 68.3% | 13.8% | 17.9% |
| **Female samples** | 3,632 | 95,249,200 | 67.7% | 15.2% | 17.1% |

*(N samples x 26,225 CpGs). Methylation categories defined by beta value cut-offs of 0.3 and 0.7.

Table S3. Summary of the global methylation across the 24,225 autosomal CpG sites (n=6,795 samples).

|  | **Min.** | **1st Quartile** | **Median** | **Mean** | **3rd Quartile** | **Max.** |
| --- | --- | --- | --- | --- | --- | --- |
| **Females (n=3,632)** | 4,029 | 6,754 | 7,135 | 7,057 | 7,368 | 11,370 |
| **Males (n=3,163)** | 3,967 | 6,770 | 7,190 | 7,053 | 7,426 | 9,464 |

Table S4. Average absolute difference in beta value by sex in CpG methylation across all 184 non cross-reactive, non-polymorphic CpG sites which passed Bonferroni Correction.

| **Min.** | **1st Quantile** | **Median** | **Mean** | **3rd Quantile** | **Max.** |
| --- | --- | --- | --- | --- | --- |
| 0.007 | 0.027 | 0.037 | 0.037 | 0.044 | 0.089 |
